# Incidence and factors associated with post-intensive care syndrome among caregivers of intensive care unit survivors: Protocol for a cohort study

**DOI:** 10.1371/journal.pone.0324013

**Published:** 2025-05-16

**Authors:** Cristobal Padilla-Fortunatti, Noelia Rojas-Silva, Sergio Cortes-Maripangue, Yasna Palmeiro-Silva, Verónica Rojas-Jara, Valentina Nilo-Gonzalez, Belén Cifuentes-Avendaño, Daniel Morales-Morales, Nicolas Garces-Brito

**Affiliations:** 1 School Nursing, Pontificia Universidad Católica de Chile, Santiago, Chile; 2 Adult Intensive Care Unit, Hospital Clínico Dra. Eloísa Diaz, Santiago, Chile; 3 Institute for Global Health, University College London, London, United Kingdom; 4 Hospital Clínico de la Universidad de Chile, Santiago, Chile; 5 Proyecto Internacional de Investigación para la Humanización de los Cuidados Intensivos (Proyecto HU-CI), Madrid, Spain; CHU Nantes, FRANCE

## Abstract

**Background:**

During the last decades, intensive care unit (ICU) mortality rates have significantly decreased but this progress has come with unintended consequences for patients and their caregivers. The adverse health-related effects observed in caregivers during the post-ICU period are referred to as Post-Intensive Care Syndrome-Family (PICS-F). Despite growing awareness of PICS-F, the long-term challenges faced by caregivers of ICU patients are not well characterized with several gaps in knowledge remaining unaddressed. The proposed study aims to determine the incidence of PICS-F impairments and identify associated factors among caregivers of ICU survivors.

**Methods:**

We plan to conduct a longitudinal prospective cohort study involving 175 caregivers of ICU patients admitted to a public hospital in Chile. Data will be collected during ICU admission, after ICU discharge, 3 months and 6 months after hospital discharge. Questionnaires will evaluate caregivers’ psychological, physical and cognitive outcomes and perceived social support, resilience, family satisfaction and caregiver burden. Factors associated with PICS-F impairments will be explored using generalised linear mixed models.

**Discussion:**

The current understanding of PICS-F is limited, particularly regarding the risk and protective factors associated with the syndrome among caregivers of ICU survivors. This study will contribute to addressing this gap by providing novel data about PICS-F and exploring previously unexamined factors linked to PICS-F such as family satisfaction, psychological buffers and caregiver burden.

**Trial Registration:**

Clinicaltrials.gov: NCT05827354. Registered on 25 April 2023.

## Introduction

The sickest patients in a hospital setting are often treated in the intensive care unit (ICU). The advances in the care of ICU patients over the last two decades have significantly decreased in-hospital mortality rates [1]. However, ICU survivors often exhibit cognitive, psychological and physical sequelae, leading to significant disability, frailty and increasing dependence on their caregivers [2,3], posing a new challenge for critical care medicine and public health [4]. Admission to an ICU is often a stressful and overwhelming experience for caregivers [5,6] due to the sudden onset of the critical illness, the risk of death of the patient and the adoption of a substitute decision-making role [7,8].

In 2010, the Society of Critical Care Medicine coined the term Post-Intensive Care Syndrome (PICS) to describe any ‘*new or worsening impairment in physical, cognitive, or mental health status arising and persisting after hospitalization for critical illness*’ to raise awareness regarding the long-term sequelae of critically ill patients [9]. An estimated 50%–70% of ICU survivors are affected by PICS [10]. The Post-Intensive Care Syndrome–Family (PICS-F) is an extension of this concept, referring to the psychological consequences that may arise after an ICU stay among caregivers [11]. However, there is limited evidence regarding the physical or cognitive impairments among caregivers of ICU survivors. PICS and PICS-F are now recognized as a public health burden with substantial associated costs [2,12]. Despite the increased awareness of PICS-F, there is a paucity of studies involving caregivers of Chilean ICU patients. Moreover, most studies have focused on describing the incidence of PICS-F, while the PICS-F-related factors are not well characterized.

## Background

### PICS-F psychological impairments

Psychological sequelae are the most frequently reported burden within PICS-F [13], with caregivers of ICU patients experiencing significant psychological distress in terms of anxiety (42%–80%), depressive symptoms (16%–90%) and symptoms of post-traumatic stress disorder (PTSD) (57%) during admission and lasting even 12 months after ICU discharge [14]. A systematic review found a wide variability in the reported incidence of anxiety (4%–94%), depressive symptoms (2%–80%) and PTSD symptoms (3%–62%) among caregivers of ICU survivors [15]. A longitudinal study with assessments at 1, 3, 6 and 12 months after ICU admission found an initial high prevalence of PTSD symptoms (36.3%) which decreased over time (24.5%, 21.5%) but increased in the last evaluation (23.6%), respectively [16]. Another longitudinal study found high levels of PTSD symptoms in 54% of caregivers with a trend to reduce during 6 months after ICU discharge [17].

During the COVID-19 pandemic, Azoulay et al. [18] reported a higher prevalence of anxiety (41% vs 34%), depressive (31% vs 18%) and PTSD (35% vs 19%) symptoms among caregivers of COVID-19 patients at 3 months after ICU discharge compared to their non-COVID-19 counterparts. Another multicenter study exploring psychological distress among caregivers of COVID-19 survivors at 3 months after ICU discharge reported anxiety, depressive and PTSD symptoms rates of 31.6%, 28.3% and 29.6%, respectively [19]. Overall, the variability in the prevalence of PICS-F psychological impairments could be attributed to the diversity of questionnaires used, cut-off values for various parameters and the timing of the assessment. Despite this heterogeneity, the prevalence of PICS-F psychological impairments among caregivers of ICU patients remains higher than the general population [15], including the Chilean population during the COVID-19 pandemic [20].

### PICS-F physical & cognitive impairments

Although not included in the original conceptualization of PICS-F [11], there are reports of physical impairments among caregivers of ICU patients. For instance, a study found a significant decline in the physical health of caregivers 6 months after hospital discharge [21]. Similarly, clinically significant fatigue was observed in 43%–53% of caregivers 4 months after hospital discharge, including worsening depressive symptoms, health risk behaviours, burden and sleep quality [22]. In the study by Fumis et al. [7], caregivers showed a decline in physical health one month after ICU discharge with a return to baseline levels at 3 months. Other studies have shown mixed results with a small trend of minimal changes in the physical health of caregivers of ICU patients [23,24].

To the best of our knowledge, there is a lack of studies exploring the potential impact of critical illness on caregivers’ cognitive function. Significant cognitive impairment is unlikely to be observed among caregivers during ICU admission since they must assume a surrogate decision-making role and frequently communicate with the ICU team [9]. Moreover, after hospital discharge, caregiving tasks are unlikely to be assumed by caregivers with relevant cognitive impairment. However, chronic exposure to psychological stress is associated with poorer cognitive function and an accelerated cognitive decline [25]. Considering the high prevalence and incidence of psychological impairments among caregivers of ICU patients, some form of cognitive impairment may be present, particularly in older caregivers.

**Fig 1:**
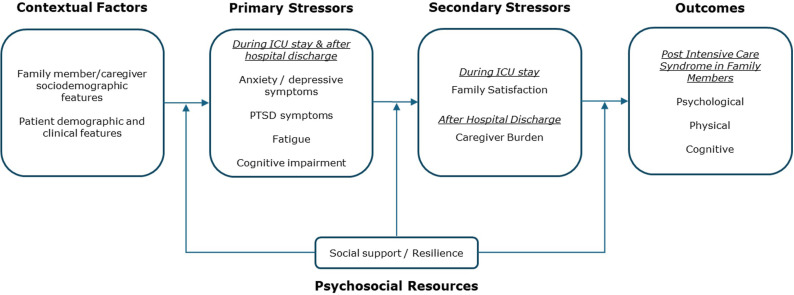
Modified version of the Caregiving Stress Model for Post Intensive Care Syndrome – Family impairments.

### Factors associated with PICS-F

Most studies have explored variables influencing PICS-F psychological impairment [13] with caregiver-related factors being younger age, female gender, lower educational level, relationship to the patient, lower financial status, history of anxiety and depression, rural residence and ethnicity [11,15]. Besides, being in a decision-making position, poor communication with the ICU healthcare team, lower educational level and having a loved one who was close to death are associated with psychological PICS-F [2]. Patient-related variables associated with PICS-F psychological impairment include severity of illness, prolonged ICU stay, older age, hospital readmission and patient disposition at discharge (e.g., home vs assisted care facility) [15]. Data on risk factors impacting physical or cognitive PICS-F impairments is scarce, with discharge to home (vs care facility) shown to be associated with significant fatigue 4 months after ICU discharge [22].

### Theoretical framework

To provide a conceptual organization of the current and potential variables that may influence known (psychological) and potential (physical and cognitive) PICS-F impairments, this study will employ an adapted version of *Pearlin’s Caregiver Stress Model* (CSM) [26]. In brief, the CSM aims to explain the occurrence of negative outcomes among caregivers (e.g., depression, anxiety and cognitive disturbances) based on the interaction of the background and contextual factors (caregiver and patient-related), primary stressors (objective and subjective indicators) and secondary strains (role and intrapsychic). Besides, the relationships between the CSM elements are influenced by psychosocial resources (coping or social support) [26]. In this study, the modified version of the CSM model for PICS-F impairments is presented in Fig 1.

The negative outcomes will be PICS-F impairments (psychological, physical or cognitive) influenced by contextual factors and primary stressors (psychological, physical and cognitive) experienced by caregivers during ICU stay and after hospital discharge. Secondary stressors can contribute to PICS-F impairments either during the ICU stay (family satisfaction) or after hospital discharge (caregiver burden). In both scenarios, psychosocial resources (social support and resilience) may buffer primary and secondary stressors and outcomes.

### Psychosocial resources

Literature pertaining to PICS-F is focused on caregivers’ psychological distress [14,15] which is described as the emotional suffering product of the imbalance between stressors and psychosocial resources [27]. Described as stress buffers or protective factors, psychosocial resources such as perceived social support, resilience or coping styles have been widely studied among caregivers of different populations [28,29] but to a lesser extent among caregivers of ICU survivors [15,30]. Perceived social support (PSS) refers to the perception of the availability of different types of resources (e.g., instrumental, psychological, informational) from the subject’s close social networks such as family or friends [31]. The few studies that have explored PSS among caregivers of ICU patients have yielded heterogeneous results. During the ICU stay, PSS showed a negative association with anxiety [32,33] and depressive symptoms [33]. In another study, PSS showed no buffer effect on perceived stress or fatigue severity [34]. Caregivers with higher PSS were found to have lower depression levels and higher psychological well-being one year after ICU discharge [23]. In the study of Azoulay et al. [18], caregivers with higher social support showed a lower risk of PTSD symptoms 3 months after ICU discharge. Conversely, a study conducted in Australia found no association between PSS and caregiver burden or self-efficacy three months after hospital discharge [35].

Resilient individuals often use different coping strategies (e.g., positive appraisal, benefit finding) to manage negative emotions related to stress [36]. Caregivers of ICU patients with high resilience may have increased mental strength that improves their ability to recover after difficulties such as the patient´s critical illness [37]. In a study of 171 caregivers, resilient caregivers exhibited fewer symptoms of anxiety, depression or PTSD during their ICU stay [38]. Similarly, another study identified resilience as a protective factor against psychological distress at ICU discharge [33]. To date, only one study has explored resilience in the post-ICU period, showing that resilient caregivers experience fewer symptoms of anxiety, depression, PTSD and caregiver burden [39].

### Family satisfaction

During a patient’s ICU stay, caregivers have specific needs that impact their experiences of the patient’s critical illness [5]. Family satisfaction (FS) refers to the extent to which healthcare providers meet these needs and expectations [40]. FS encompasses key aspects of the ICU experience such as communication with healthcare providers, emotional support, proximity to the patient, ICU environment, decision-making involvement and nursing care of the patient [41]. FS may influence post-ICU outcomes such as the psychological dimension of the PICS-F. In a small-scale study, FS and communication with ICU staff showed a negative association with depressive and PTSD symptoms 2 months after ICU discharge [42]. Likewise, low levels of FS during ICU stay were associated with higher levels of anxiety, depression and PTSD symptoms 3 months after hospital discharge [43]. Higher FS with care and decision-making involvement were found to be associated with a lower risk of depression 3 months after ICU admission [44]. Although the role of FS in other PICS-F impairments (e.g., physical, cognitive) is unclear, meeting the needs of caregivers during and after their stay in the ICU may decrease the negative impact of PICS-F [45].

### Caregiver burden

Up to 80% of the ICU caregivers become informal caregivers after the patient’s hospital discharge [45,46]. Caregiving is often described as a stressful process that can lead to a multidimensional burden. Caregiver burden (CB) comprises the physical, psychological, social and financial problems associated with the care of an impaired person [47]. CB has been extensively studied in caregivers of patients with different conditions [48] but to a lesser extent in caregivers of ICU survivors. During the ICU stay, caregivers often assume a partial caregiving role such as providing emotional support or surrogate decision-making [11]. However, after hospital discharge, frailty and dependency may arise leading to an increase in caregiving tasks [14].

In a study, 16% of caregivers experienced a high CB and the high CB was associated with anxiety and depressive symptoms [49]. In another study, 53% of caregivers experienced high CB with a positive association with symptoms of anxiety, depression and PTSD as well as sleep disturbances and poor quality of life [50]. Comini et al. [51] reported a high proportion of caregivers with critical levels of burden. Remarkably, although survivors’ global condition improved 6 months after discharge, caregiver strain remained high and the percentage of caregivers needing support tended to increase. Another study found a positive association between CB and depressive symptoms 3 months after ICU admission [52]. In addition, a higher CB was shown to be associated with worse mental health and an elevated risk of psychological symptoms 3 months after ICU discharge [53]. Although the association between CB and PICS-F psychological impairment among caregivers of ICU survivors has been reported, the relationship with caregiver-related variables measured during ICU stay (e.g., psychological distress) remains unclear. Furthermore, a study of the potential association between CB and PICS-F physical and cognitive impairments is warranted due to the conceptual overlap between CB and PICS-F.

## Materials and methods

### Aims, design, and setting

The primary aim of this study is to determine the incidence of PICS-F impairments (psychological, physical and cognitive) among caregivers of ICU survivors of a public hospital in Chile. A secondary aim is to identify factors during ICU stay and after hospital discharge, associated with the PICS-F impairments. To achieve these aims, a prospective analytical cohort study will collect data from ICU caregivers between the 3^rd^ and 10^th^ day of ICU admission (T_1_), after ICU discharge (T_2_) and 3 months (T_3_) and 6 months (T_4_) after hospital discharge. This study will follow the STROBE guidelines for reporting observational cohort studies [54].

The study will be conducted in a polyvalent 24-bed adult ICU belonging to the second largest high-complexity public hospital in the Southeast area in Santiago, Chile, with around 400 beds available. The ICU’s visiting policy allows for a 1–2-hour daily visit with flexibility in exceptional cases. Concerning ICU personnel composition, the nurse-to-patient ratio is 1:3 while the medical staff is composed of 4 residents a day plus 3 senior ICU physicians. Daily updates regarding the patient´s condition are provided to the responsible family member (caregiver) by residents during visiting hours but in complex cases (e.g., end of life) a senior ICU physician is involved. Regarding other professionals, physiotherapists are available as well as a social worker who performs mostly administrative tasks related to family members.

### Inclusion and exclusion criteria

All adult caregivers identified as a patient’s representative and likely to become responsible for providing direct or indirect care after hospital discharge will be eligible for inclusion. In addition, the inclusion criteria for patients will be as follows: age > 18 years; ICU length of stay between 3 and 10 days; and receiving positive pressure respiratory support. Caregivers of ICU patients with an impending death risk or likely to be discharged from the ICU will be excluded. Caregivers will be withdrawn from the study if the patient dies at any point during the study and no further data will be collected.

### Sample size and power

Sample size was calculated based on the primary aim of estimating the incidence of PICS-F impairments. We aimed for a precision level (*e*) of 5% (representing the half-width of the 95% confidence interval) around the expected incidence estimate. Due to the lack of studies utilizing identical questionnaires and cut-off values in a comparable population, we conservatively used an expected incidence (*P*) of 20% of PTSD symptoms reported by Heesakkers et al. [19]. Using a 95% confidence level (corresponding to *Z* = 1.96, alpha = 5%), the required sample size was calculated using the formula: n = [*Z²* * *P* * (1-*P*)]/ *e²*, where *P* is the expected true proportion in the population and *e* is the desired precision level. Consequently, the initial sample size required was 247 caregivers.

Then, the estimated total population of eligible subjects that could meet our inclusion criteria before the projected recruitment start was approximately 600 caregivers. Then, the initial sample size of 247 represented 41.2% of this estimated finite population, exceeding the commonly used threshold of 5–10%. Consequently, a finite population correction was applied using the formula [55]: Adjusted n = (finite population * initial sample size)/ (finite population + initial sample size). Thus, the adjusted sample size will be 175 caregivers. However, factoring in a potential 40% attrition rate reported in the literature [53,56], 292 caregivers will be targeted for enrolment.

### Study timeline and variables

**Fig 2:**
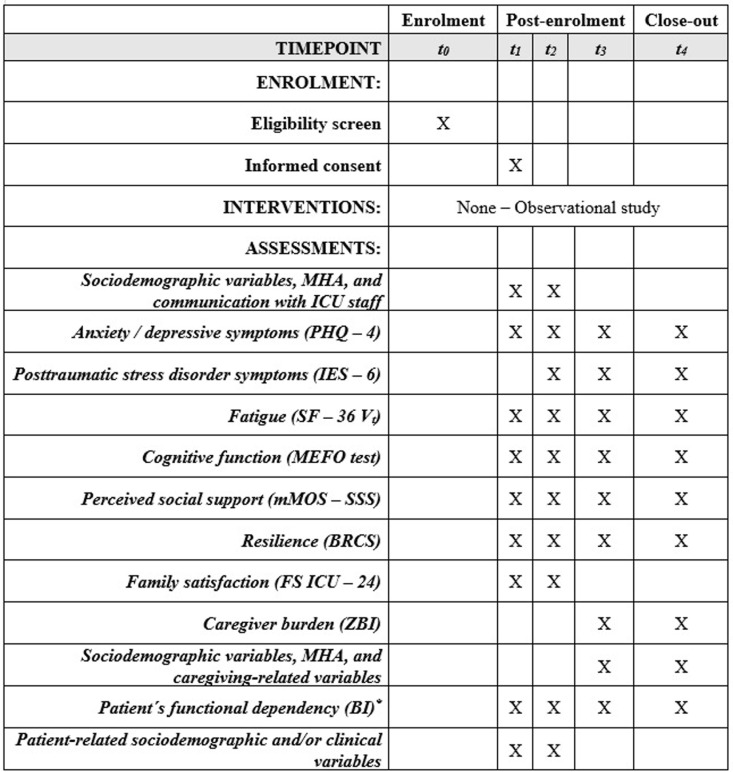
SPIRIT schedule: Timeline of recruitment and assessment. *t*_0_: Daily screening; *t*_1_: Between the 3^rd^ and 10^th^ day of ICU admission; t_2_: Near after ICU discharge; *t*_3_: 3 months after hospital discharge; *t*_4_: 6 months after hospital discharge. Abbreviations: ICU Intensive Care Unit; MHA: Mental Health Antecedents; PHQ-4: *Patient Health Questionnaire – 4*; IES – 6: *Impact of Event Scale – 6*; SF – 36 V_t_: *Short Form Health Survey – 36 Vitality*; MEFO: *Memory, Fluency, and Orientation*; mMOS – SSS: *Modified Medical Outcomes Study Social Support Survey*; BRCS: *Brief Resilient Coping Scale*; *Family Satisfaction with Care in the Intensive Care Unit – 24*; ZBI: *Zarit Burden Interview*; BI: Barthel Index. * Will be completed by the caregiver at *t*_*1*_ regarding the patient’s functional independence status before ICU admission. At *t*_*2*_*, t*_*3*_*,* and *t*_4_ it will be applied regarding the current patient status.

Relevant sociodemographic variables of caregivers plus demographic and clinical data of the patients will be collected at each time point. Also, patients’ demographic and clinical data will be acquired from their medical charts. The schedule presented in Fig 2 describes the timeline of enrolment and assessments.

### Questionnaires

At each time point, a set of different validated screening questionnaires (Table 1) will be used to explore the incidence of each PICS-F impairment and the role of psychosocial resources, family satisfaction and caregiver burden.

**Table 1:** Questionnaire description.

Measure	N° of items	Score range	Scoring and interpretation	Cut-off value	PICS-F dimension
Patient Health Questionnaire for Depression and Anxiety (PHQ–4) [20,57]	4	0 - 12	Sum of all items. Higher scores reflect higher levels of anxiety/depressive symptoms.	≥ 6	Psychological
Impact of Event Scale-6 (IES-6) [58,59]	6	0 - 4	Average of all items. Higher scores suggest a higher level of PTSD symptoms.	≥ 1.75	Psychological
Modified Medical Outcomes Study Social Support Survey (mMOS-SSS) [60,61]	8	0 - 100	Average of all items. Higher scores reflect higher levels of perceived social support.	N/A	N/A
Brief Resilient Coping Scale (BRCS) [62,63]	4	4 - 20	Sum of all items. Higher scores reflect higher resilience	≤ 13	N/A
Memory, fluency, and orientation (MEFO) test [64]	3	0 - 13	Sum of all items. Higher scores reflect a lower degree of cognitive impairment.	< 9	Cognitive
Energy/vitality subscale of the Short Form Health Survey (SF–36 Vt) [22,65,66]	4	0 - 100	Average of all items. Lower scores reflect lower levels of vitality (fatigue).	< 45	Physical
Family Satisfaction with Care in the Intensive Care Unit–24 (FS ICU–24) [67,68]	24	0 - 100	Average of all items. Higher scores indicate higher levels of satisfaction.	N/A	N/A
Zarit Burden Interview (ZBI) [69,70]	7	5 - 35	Sum of all items. Higher scores reflect a higher level of burden.	≥ 17	N/A
Barthel Index (BI) [71,72]	10	0 - 100	Sum of all items. Higher scores reflect higher functional independence.	N/A	N/A

Abbreviations. PICS-F: Post Intensive Care Syndrome Family; PTSD: Posttraumatic stress disorder; N/A: Not Applicable.

### Data collection and management

A dual (patient/caregiver) screening process will be conducted daily by a collaborating ICU nurse to confirm eligibility based on inclusion/exclusion criteria. During visiting hours, a research assistant will assess caregivers´ eligibility and approach them if they appear emotionally stable. If interested, caregivers will sign the consent form and complete the initial survey (T_1_), either in person or by phone. Subsequent data collection (T_2,_ T_3_, T_4_) will be performed via phone calls. Participant caregivers will be financially compensated for their time spent filling out the questionnaires at T_2_ and T_4_.

Caregiver and patient-related data will be anonymized to ensure privacy. Each participant will be assigned a unique identifier to prevent the identification of personal or questionnaire response data. All information will be kept safe in a password-protected database, and access will be granted to the study team. Data monitoring and auditing will be performed by the principal investigator and study coordinator.

### Outcomes and statistical analyses

Descriptive statistics will include summary statistics and plots to explore and characterize data. Normally distributed continuous variables will be reported as mean ± standard deviation and non-normally distributed variables as median (interquartile range). Categorical variables will be reported as frequency (percentage). For each questionnaire, internal consistency will be assessed by calculating Cronbach’s alpha.

To determine the incidence of each PICS-F impairment, four dichotomous dependent variables will be defined: psychological (PHQ-4 ≥ 6 or IES-6 ≥ 1.75), physical (SF-36 V_T_ < 45) and cognitive (MEFO < 9) impairments at T_3_ and T_4_. Subjects scoring above or below the respective cut-off value will be categorized as having PICS-F in their respective impairment at T_3_ and/or T_4_. The number of subjects showing a given PICS-F impairment will be divided by the total number of subjects at T_3_ and T_4_. Then, bivariate and multivariate analyses will be conducted to identify factors associated with PICS-F impairments. For the multivariate generalised linear mixed models (GLMMs), variable selection will be guided by established theoretical frameworks, prior literature findings, and clinical relevance. To further enhance model robustness, prevent overfitting, and handle potential multicollinearity, we will consider employing penalised regression techniques, such as LASSO (Least Absolute Shrinkage and Selection Operator) or elastic net, within the GLMM framework.

Longitudinal analysis will be performed to identify variables associated with the PICS-F impairments over time. First, graphical methods, such as panel line plots, will be used to visualize the trend and inter-subject and intra-subject variation over time. This analysis will be useful for determining important covariates and the regression models to be included and applied afterwards. Second, the strength of the outcome correlation should be defined to decide the regression methods. In this scenario and considering the nature of the outcome, generalized linear mixed models (GLMM) will be used [73,74]. Contextual factors (caregiver and patient variables) of each participant will be considered fixed effects, but random effects will be included because of the expected variation in the outcome at each time point and for each subject. Given that the outcomes are measured as a dichotomous variable, the family will be binomial and the link function is expected to be logit. Then, model building will consider the main effects and interactions based on theoretical models, which will be evaluated through Akaike Information Criterion or Bayesian Information Criterion. The initial models proposed for this study are presented in Table 2.

**Table 2:** Variables and generalized linear mixed models to explore PICS-F impairments.

	Independent Variables								Dependent Variables			
Model #	T_1_ - ICU admission								T_3_ & T_4_^a^			
	*Patient*	*Family member*	*PHQ-4*	*IES-6*	*mMOS -SSS*	*BRCS*	*SF-36 VT*	*MEFO*	*PHQ-4*	*IES-6*	*SF-36 VT*	*MEFO*
1	Age, SOFA, COVID-19 diagnosis, days since ICU admission, BI	Age, gender, education, kinship, MHA, employment status	X		X	X	X	X	X			
2			X		X	X	X	X		X		
3			X		X	X	X	X			X	
4			X		X	X	X	X				X
	T_2_ - After ICU discharge								T_3_ & T_4_^a^			
	*Patient*	*Family member*	*PHQ-4*	*IES-6*	*mMOS-SSS*	*BRCS*	*SF-36 VT*	*MEFO*	*PHQ-4*	*IES-6*	*SF-36 V*_*T*_	*MEFO*
5	Age, COVID-19 diagnosis, ICU LOS, BI	Age, gender, education, kinship, MHA, employment status	X	X	X	X	X	X	X			
6			X	X	X	X	X	X		X		
7			X	X	X	X	X	X			X	
8			X	X	X	X	X	X				X
	T_3_ - 3 months after hospital discharge								T_3_			
	*Patient*	*Family member*	*PHQ-4*	*IES-6*	*mMOS-SSS*	*BRCS*	*SF-36 VT*	*MEFO*	*PHQ-4*	*IES-6*	*SF-36 V*_*T*_	*MEFO*
9	Age, COVID-19 diagnosis, ICU and hospital LOS, hospital readmission, discharge location, BI	Age, gender, education, kinship, MHA, employment status, caregiving hours		X	X	X	X	X	X			
10			X		X	X	X	X		X		
11			X	X	X	X		X			X	
12			X	X	X	X	X					X
									T_4_			
13			X	X	X	X	X	X	X			
14			X	X	X	X	X	X		X		
15			X	X	X	X	X	X			X	
16			X	X	X	X	X	X				X
	T_4_ - 6 months after hospital discharge								T_4_			
	*Patient*	*Family member*	*PHQ-4*	*IES-6*	*mMOS-SSS*	*BRCS*	*SF-36 VT*	*MEFO*	*PHQ-4*	*IES-6*	*SF-36 V*_*T*_	*MEFO*
17	Age, COVID-19 diagnosis, ICU and hospital LOS, hospital readmission, BI, discharge location	Age, gender, education, kinship, MHA, employment status, caregiving hours		X	X	X	X	X	X			
18			X		X	X	X	X		X		
19			X	X	X	X		X			X	
20			X	X	X	X	X					X

^a^Models will be tested with T_3_ and T_4_ dependent variables separately. Abbreviations: SOFA: sequential organ failure assessment score; ICU: intensive care unit; BI: Barthel index; MHA: mental health antecedents; LOS = length of stay; PHQ-4: patient health questionnaire; IES-6: impact of event scale; mMOS-SSS: modified medical outcomes study social support scale; BRCS: brief resilience coping scale; SF-36 V_T_: short-form health survey, vitality subscale; MEFO: memory, fluency, and orientation test.

Then, if FS or CB is significantly associated with any of PICS-F impairments at T_3_ and/or T_4_, GLMM with and without the selected secondary stressor will be fitted to analyse the extent of this association. First, FS measured at T_1_ will be added to models 1–4 while the FS measured at T_2_ will be included in models 5–8. Similarly, CB measured at T_3_ will be included in models 9–20 while the T_4_ measure of CB will be added to models 17–20. The original model without a secondary stressor will be contrasted with the same model plus the secondary stressor (either FS or CB). Also, 95% confidence intervals will be reported and a *p* < 0.05 will be considered statistically significant. All statistical analyses will be conducted in R, using the applicable packages [75].

### Missing data

Given the longitudinal study design, some missing data is anticipated. Missing data patterns will be examined to determine if they are missing at random or not missing at random. Depending on the result, appropriate models for estimation will be applied (i.e., selection or pattern mixture models or predictive mean matching) and sensitivity analyses will be conducted.

### Ethical considerations

This study will comply with the principles of the Helsinki [76] and Singapore Declarations [77]. Ethical approval for this study was obtained from the Health Sciences Ethics and Scientific Committee at the Pontificia Universidad Catolica de Chile (April 2023) and Southeast Metropolitan Health Service (June 2023). All participating caregivers will sign in-person the informed consent form. Ethics Committees have waived informed consent for acquiring patient-related data from medical charts. Changes to the study protocol and/or the informed consent form will be sent to respective ethics committees as protocol amendments (S1-S2 Appendix).

### Status of study

Recruitment of participants began on June 30, 2023. As of January 23, 2025, 223 participants had been enrolled, completing baseline measures and planned follow-ups whenever they proceed. Participant recruitment is expected to conclude around June 2025.

## Discussion

The decline in ICU mortality rates over recent decades has led to a growing focus on PICS, with researchers highlighting several challenges for healthcare systems and society, particularly for caregivers of critical illness survivors. Nonetheless, the long-term issues faced by caregivers of ICU patients, including PICS-F, remain poorly understood [78]. To the best of our knowledge, this is one of the first longitudinal studies in Chile to formally investigate the incidence and factors related to PICS-F. Furthermore, it will be one of the first to comprehensively explore physical and cognitive impairments in addition to psychological PICS-F impairments, utilizing the CSM framework [26]. Although Cameron et al. [23] employed the CSM, their study did not measure FS as a secondary stressor or resilience as a psychological resource and they did not explore potential cognitive impairments.

Despite growing awareness of PICS-F, physical and cognitive impairments remain understudied. This study will address this gap by investigating the incidence and factors associated with PICS-F physical impairment and exploring caregivers’ cognitive function during and after ICU stay. The scientific novelty of this study is also grounded in the inclusion of CB as a secondary stressor that may impact PICS-F impairments. While evidence of CB in caregivers of ICU survivors is emerging, this study recognizes that the PICS-F impairments may not solely result from the residual effect of a traumatic event such as an ICU admission, but also from the ongoing burden of caring for an ICU survivor.

### Limitations

There are some foreseeable limitations of the proposed study. First, the single-centre scope of the study limits the generalizability of our findings to caregivers of ICU survivors with similar features. Future multicentric studies, including bereaved family members, are required to understand the role of the novel PICS-F variables explored in this study. Second, the sole utilization of questionnaires for data collection, which have well-known limitations (e.g., recall bias), should be complemented in future studies with objective measures while available. Third, limited visiting hours available may influence the caregiver´s psychological symptoms. Fourth, the longitudinal design may increase the odds of attrition and/or withdrawal of participants. Similarly, the low recruitment/high loss to follow-up rates observed among similar studies [18,23] may lead to selection bias, particularly in those caregivers who will not be approached because of evident psychological distress.

## Conclusion

ICU survivorship imposes a significant burden on the healthcare system, society and in particular, caregivers of those who endure a critical illness. The proposed study of caregivers of ICU survivors at a public hospital in Chile aims to provide comprehensive insights into the incidence and factors associated with PICS-F impairments. By examining PICS-F-related factors during ICU stay and after hospital discharge, this study will inform healthcare stakeholders and researchers, enabling the development of tailored interventions that address the full spectrum of critical illness, from ICU admission to the caregiving period. This will ultimately improve our understanding of PICS-F and help enhance support for caregivers and ICU survivors.

## Supporting information

S1 Appendix Checklist. SPIRIT 2013 checklist: Recommended items to address in a clinical trial protocol and related documents.
(DOC)

S2 Appendix Original protocol sent to ethics committee. Original protocol sent to ethics committee (in English).
(PDF)
