# Supplementary material for: Incidence and factors associated with post-intensive care syndrome among caregivers of intensive care unit survivors: Protocol for a cohort study
Source: PLoS One. 2025 May 16;20(5):e0324013. doi: 10.1371/journal.pone.0324013 (PMC12083806; doi:10.1371/journal.pone.0324013)
Supplement: S2 Appendix — Original protocol sent to ethics committee (in English). (PDF) [file pone.0324013.s002.pdf]

INCIDENCE AND FACTORS ASSOCIATED TO THE DEVELOPMENT OF POST-INTENSIVE CARE SYNDROME AMONG FAMILY MEMBERS OF INTENSIVE CARE UNIT SURVIVORS: A LONGITUDINAL EXPLORATORY STUDY

BACKGROUND

The care and treatment of the sickest patients in a hospital setting are often performed within an intensive care unit (ICU). Life-threatening conditions can lead to mortality rates between 15-20% among ICU patients.[1] Nonetheless, the technical and scientific evolution in the care of ICU patients over the last two decades has radically improved the clinical practice with a decrease in-hospital mortality rates.[2] This increase in survivorship, however, has been associated with several cognitive, psychological, and physical impairments among ICU patients after discharge.[3] Former critically ill patients can experience serious disability and frailty, leading to increased dependence on their caregivers.[4] ICU survivorship has become a new challenge for critical care medicine and public health.[5]

For family members, admission to an ICU is often a stressful and overwhelming experience[6,7] due to the sudden onset of the critical illness, the risk of death of the patient, and the adoption of a substitute decision-making role.[8,9] Besides, other stressors may arise for family members such as an unfriendly technological environment, restrictive visitation policies, and low-quality communication with ICU staff.[10,11] In this scenario, family members may experience significant psychological distress in terms of anxiety (42-80%), depressive (16-90%), and posttraumatic stress disorder (PTSD) symptoms (57%) during admission and may persist, along with other negative outcomes, even 12 months after ICU discharge.[1]

In 2010, the Society of Critical Care Medicine coined the term Post Intensive Care Syndrome (PICS) to refer to any "*new or worsening impairment in physical, cognitive, or mental health status arising and persisting after hospitalization for critical illness*" to raise awareness of the growing body of evidence regarding the long-term sequels of critical illness.[12] PICS prevalence is estimated to affect between 50-70% of ICU survivors.[13] As an extension of this construct, the Post Intensive Care Syndrome – Family (PICS-F) comprises psychological, physical, cognitive impairments, and social consequences that may arise after an ICU stay among family members.[14] Both PICS and PICS-F are now being recognized as a public health burden with substantial associated costs.[3,15] Despite the increased awareness in PICS-F, evidence to date has focused on the incidence of PICS-F impairments and to a lesser extent, to explore PICS-F related factors. A summary of the evidence on each of the PICS-F impairments, social consequences, risk factors, interventions, and proposed novel factors to be explored in this study is presented below:

PICS-F Psychological Impairments

Within PICS-F impairments, psychological sequelae are the most investigated[16] and have been associated with the use of psychotropic medication in up to 32% of family members who start these medications after ICU admission and 40% seeking healthcare professionals for emotional problems.[1] In a systematic review including 22 studies published until 2018, family members of ICU survivors reported a variable incidence of anxiety (4%-94%), depressive (2%-80%), and PTSD (3%-62%) symptoms after ICU discharge.[17] The only published study conducted in Chile showed an incidence of 22.9% of PTSD symptoms 2 months after hospital discharge, which resembles subjects who endured the 2010 earthquake in Chile.[18]

Recently published studies have shown similar results. In a single-center study, family members reported 30.6% of anxiety symptoms, 25% of depressive symptoms, and 11% of PTSD symptoms 2 months after ICU discharge.[19] In a small study involving 62 family members, PTSD symptoms were highly prevalent (69.4%) 3 months after ICU discharge.[20] In a multicentric study, Milton et al.[21] reported a prevalence of 8% of anxiety symptoms, 15% of depressive symptoms, and 21% of PTSD symptoms among family members 3 months after ICU discharge. In a longitudinal study with measures at 1, 3, 6, and 12 months after ICU admission, an initial high prevalence of PTSD symptoms (36.3%) decreased over time (24.5%, 21.5%) but increase in the last evaluation (23.6%).[22] Another longitudinal study that involved 221 family caregivers found high levels of PTSD symptoms in 54% of the sample with a trend to reduction during the first 6 months after ICU discharge.[23]

During the current COVID-19 pandemic, Azoulay et al.[24] reported a higher prevalence of anxiety (41% vs 34%), depressive (31% vs 18%), and PTSD (35% vs 19%) symptoms were observed 3 months after ICU discharge among family members of COVID-19 patients, compared to their non-COVID-19 counterparts. In another multicentric study, anxiety, depressive, and PTSD symptoms were experienced at 3- and 12-months post ICU discharge by 31.6% - 29%, 28.3% - 22.8%, and 29.6% - 20.2% of family members of COVID-19 survivors respectively.[25] Overall, the variability in the prevalence of PICS-F psychological impairments could be attributed to the diversity of questionnaires utilized, cut-off values to

establish caseness, and the specific moment in which the questionnaires were applied. Despite this heterogeneity, the prevalence of PICS-F psychological impairments remains higher than in the general population,[17] including the Chilean population during the COVID-19 pandemic.[26]

PICS-F Physical & Cognitive Impairments

Compared to PICS-F impairments, physical impairments among family members of ICU survivors have received little attention. Among family members of former ICU ventilated patients, a significant decline in physical health was reported 6 months after hospital discharge.[27] Similarly, clinically significant fatigue was observed in 43%-53% of ICU family members 4 months after hospital discharge. Likewise, these caregivers reported a worsening in depressive symptoms, health risk behaviors, burden, and sleep quality.[28] In the study of Fumis et al.,[8] family members showed a decline in physical health 1 month after ICU discharge with a return to baseline levels at 3 months. Other studies have shown mixed results with a small trend to minimal changes in the physical health of family members of ICU survivors.[29,30]

Although cognitive impairment is common among ICU survivors and ranges from 30% to 80% after hospital discharge,[31] studies exploring the potential impact of critical illness on family members' cognitive function are missing. Significant cognitive impairment is unlikely to be prevalent among family members during ICU admission since they must assume a surrogate decision-making role and frequently communicate with the ICU team.[12] Furthermore, after hospital discharge caregiving tasks are unlikely to be assumed by a relative with relevant impairment in cognition. However, chronic exposure to psychological stress is associated with poorer cognitive function and an accelerated cognitive decline.[32] Considering the high prevalence of psychological impairments among family members of ICU survivors, some form of cognitive impairment may be present, particularly in older family members of ICU survivors.

PICS-F Socioeconomic Consequences

Despite not being included in the PICS/PICS-F original definitions, socioeconomic burden during and after a critical illness has been described among family members of ICU survivors.[33] Socioeconomic consequences among family members of ICU survivors have been described in terms of loss of employment, financial issues/burden, lifestyle interference, challenge to family dynamics, exacerbation of chronic health conditions, and a decrease in their health-related quality of life.[1,12] In a small study, 70% of family members expressed some form of financial worry during and after ICU discharge.[33] Likewise, in the study of Swoboda and Lipsett,[34] 45% of family members reported economic issues related to basic needs, housing, and medical care. Moreover, 1 year after ICU discharge, 36.7% of these family members had to move to a less expensive place, delay educational plans or medical treatment, leading to bankruptcy. In secondary data analysis, 48.5% of the family members reported serious financial stress at 3 months and 38.5% at 6 months after ICU discharge with a direct effect on symptoms of anxiety and depression.[35]

Factors Associated with PICS-F

Despite being a multidimensional construct, PICS-F impairments (psychological, physical, and cognitive) have been mostly explored separately with psychological impairment-related factors, being the most reported.[16] Family member-related factors for PICS-F psychological impairments include younger age, female gender, lower educational level, relationship to the patient, lower financial status, history of anxiety and/or depression, rural residence, and ethnicity.[14,17] Besides, being in a decision-making position, poor communication with the ICU health team, lower educational level, and having a loved one who was close to death have been associated with PICS-F.[3] Concerning patient-related variables, severity of illness, prolonged ICU stay, older age, hospital readmission, and patient disposition at discharge (e.g., home vs assisted care facility) have been reported to be associated with PICS-F psychological impairment.[17] Data on risk factors impacting remaining PICS-F impairments is scarce with discharge to home (vs care facility) being associated with significant fatigue (PICS-F physical) 4 months after ICU discharge.[28]

PICS-F Interventions

Regardless of the awareness of the increased impact and prevalence of the PICS-F, studies reporting interventions are few with heterogeneous results.[36] Furthermore, they have exclusively focused on PICS-F's psychological impairments. Interventions conducted in the post-ICU period are noticeably less than those delivered during the ICU stay. For instance, a multidimensional rehabilitation manual with an emphasis on relaxation and coping strategies plus biweekly phone calls showed no effect on anxiety, depressive, or PTSD symptoms neither at 2 nor at 6 months after ICU discharge.[37] Likewise, the provision of emotional and instrumental support, as well as instrumental case management services, reported no improvements at 2 months post-hospital discharge in caregiving burden, depressive symptoms, or physical health.[38]

Recently published interventions have shown a trend towards positive results. In a before-and-after study, the participation of family members in patient care showed a significant decrease in PTSD symptoms but no effect on depressive symptoms 3 months after ICU discharge.[39] A pilot intervention based on the delivery of cognitive-behavioral therapy via a smartphone app reduced PTSD symptoms in ICU family members 2 months after discharge.[40] Family members' participation in the writing of a diary for the ICU patient showed no significant differences in anxiety, depressive, and PTSD symptoms 3 months after ICU discharge.[41] To summarize, the dissimilar effectiveness observed in PICS-F interventions, particularly those centered on psychological impairments, suggests that PICS-F is a construct that is still not been fully understood and relevant factors may be missed.

Psychosocial Resources

PICS-F literature is focused on family members' psychological distress.[1,17] Nonetheless, psychological distress can be described as the emotional suffering product of the imbalance between stressors and psychosocial resources.[42] Described as stress buffers or protective factors, psychosocial resources such as perceived social support, resilience, or coping styles have been widely studied among caregivers of different populations[43,44] but to a lesser extent among caregivers of ICU survivors.[17,45]

Perceived social support (PSS) refers to the perception of the availability of the different types of resources (e.g., instrumental, psychological, informational) by the subject´s close social networks such as family or friends.[46] Different mechanisms are proposed to explain PSS buffer properties on stress. First, the perception that others can deliver the required resources could result in appraising a situation as less stressful. Second, those who receive support may buffer the impact of stress by providing solutions to problems, decreasing the apparent relevance of the problem, by giving a distraction from the problem, or by contributing to healthy behaviors.[47]

Studies exploring PSS among ICU family members are limited with heterogeneous results. During the ICU stay, PSS has been negatively associated with anxiety[48,49] and depressive symptoms.[49] On the contrary, PSS showed no buffer effect on perceived stress or fatigue severity.[50] Then, ICU family members with higher PSS informed lower depression levels, higher psychological well-being, and mental health 1 year after ICU discharge.[29] In the study of Azoulay et al.[24], family members with higher satisfaction with their social support reported a lower risk to develop PTSD symptoms 3 months after ICU discharge (OR= 0.82, 95% CI 0.74-0.90). Conversely, in a sample of 71 caregivers of ICU survivors in Australia, PSS was not associated with caregiver burden nor self-efficacy 3 months after hospital discharge.[51]

Next, resilient individuals often use different coping strategies (e.g. positive appraisal, benefit finding) to manage negative emotions related to stress.[52] ICU family members with high resilience may have an increased mental strength that improves their ability to recover after difficulties such as the patient´s critical illness.[53] In a study involving 171 family members, fewer symptoms of anxiety, depression, or PTSD were observed among resilient family members during their ICU stay.[54] Similarly, resilience was found to be a protective factor against psychological distress at ICU discharge.[49] To date, only one study has explored resilience in the post-ICU period showing that resilient family members of ICU survivors experienced fewer symptoms of anxiety, depression, PTSD, and caregiver burden after ICU discharge.[55] The limited evidence to date suggests a protective role of resilience during the ICU stay of family members that should be explored on PICS-F impairments, after hospital discharge.

Family Satisfaction

During the ICU stay of the patient, family members experience a specific group of needs that influence their experiences of the patient's critical illness.[6] Family satisfaction is the extent to which the needs and expectations of family members are met by healthcare providers during their ICU stay.[56] As a complex and multidimensional construct, family satisfaction comprises the evaluation by family members of key elements of the ICU experience such as communication with healthcare providers, emotional support, closeness to the patient, ICU environment, decision-making involvement, and nursing care of the patient.[57]

A negative association has been reported between family satisfaction and symptoms of anxiety and depression during ICU stay.[10,58] Moreover, some studies have shown that a worst ICU experience (measured as family satisfaction) may influence post-ICU/hospital outcomes such as PICS-F´s psychological impairments. In a small study, family satisfaction and communication with ICU staff were negatively associated with depressive and PTSD symptoms 2 months after ICU discharge. Likewise, low levels of family satisfaction during ICU stay have been associated with higher levels of anxiety, depressive, and PTSD symptoms 3 months after hospital discharge.[59] Then, higher levels of key family satisfaction attributes such as satisfaction with care and decision-making involvement were associated with less risk of depression 3 months after ICU admission.[60] Although the role of family satisfaction in other PICS-F

impairments (e.g., physical, cognitive) is unknown, meeting the needs of family members during and after their stay in the ICU could decrease the negative impact of PICS-F on family members as caregivers.[61]

Caregiver burden

Up to 80% of the ICU family members become informal caregivers after the patient's hospital discharge.[61,62] Caregiving is often described as a stressful process that can lead to a significant and multidimensional burden. Caregiver burden (CB) comprises the physical, psychological or emotional, social, and financial problems associated with the care of an impaired person.[63] CB has been extensively studied in caregivers of patients with different conditions[64] but to a lesser extent on family members who provide care to ICU survivors. During the ICU stay of the patient, family members assume a partial caregiving role such as emotional support or surrogate decision-making.[14] However, after hospital discharge, frailty and dependency may arise leading to an increase of caregiving tasks.[1]

Current evidence suggests that being a caregiver of an ICU survivor imposes an elevated burden and strain on family members. In one study, 16% of caregivers reported a high CB and it was positively associated with anxiety and depressive symptoms, but not with PTSD symptoms.[65] In another study, 53% of caregivers experienced high CB with a positive association with anxiety, depressive, and PTSD symptoms as well as with sleep disturbances and poor quality of life.[66] Comini et al.[67] reported a high percentage of caregivers with critical levels of burden. Remarkably, although survivors' global condition improved 6 months after discharge, caregiver strain remained high and the percentage of caregivers needing support tended to increase. In another study, a positive association between CB and depressive symptoms was reported 3 months after ICU admission.[68] Also, a higher CB has been associated with worse mental health and an elevated risk of psychological symptoms 3 months after ICU discharge.[21] Although the association between CB and PICS-F psychological impairment on caregivers of ICU survivors has been reported, the relationship with family member-related variables measured during ICU stay (e.g., psychological distress) remains unclear. Besides, the study of a potential association between CB and PICS-F physical and cognitive impairments is warranted due to the possible conceptual overlap between CB and PICS-F.

Theoretical Framework

To provide a conceptual organization of the current and potential variables that may be influencing PICS-F impairments, this study will employ an adapted version of the *Pearlin´s Caregiver Stress Model* (CSM).[69] In brief, the CSM aims to explain the occurrence of negative outcomes among caregivers (e.g., depression, anxiety, cognitive disturbances, etc.) based on the interaction of the background and contextual factors (family member and patient-related), primary stressors (objective and subjective indicators), and secondary strains (role and intrapsychic). Besides, the relationships between the CSM elements are mediated by psychosocial resources (coping or social support).[69]

In this study, the negative outcomes will be PICS-F impairments (psychological, physical, or cognitive) that are influenced by contextual factors and primary stressors (psychological, physical, and cognitive) experienced by family members during ICU admission/stay and after hospital discharge. Then, secondary stressors can contribute to PICS-F impairments either during the ICU stay (family satisfaction) or after hospital discharge (caregiver burden). In both scenarios, psychosocial resources (social support and resilience) may buffer the primary stressors, secondary stressors, and outcomes. Figure 1 presents the proposed modified version of the CSM model for PICS-F impairments for this study.

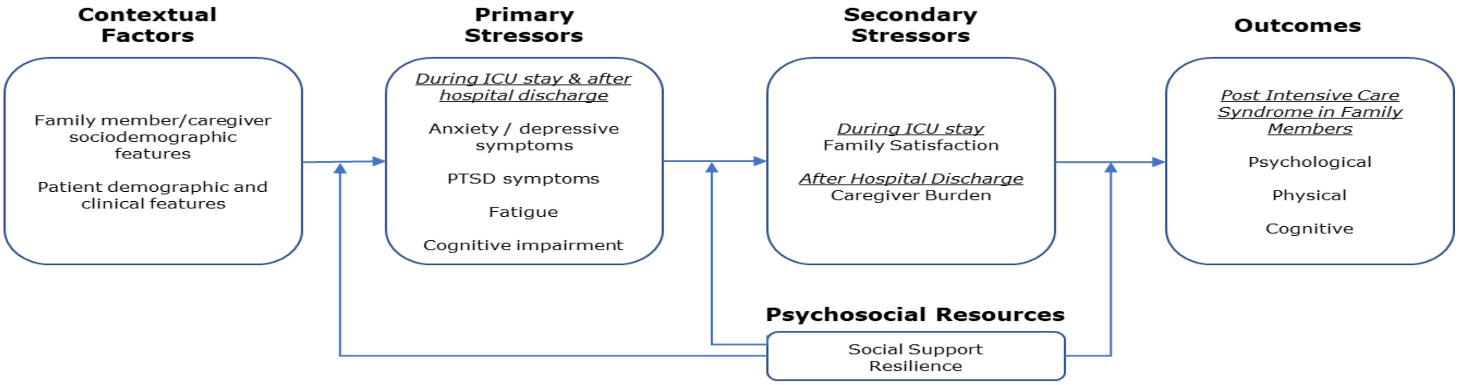

Figure 1. Caregiving Stress Model[69] adapted to Post Intensive Care Syndrome – Family impairments.

Research questions: The research questions that will guide the study are: In a sample of family members of ICU survivors of a public hospital in Chile:

1) what is the incidence rate of each PICS-F impairment at three and six months after hospital discharge?
2) what are the contextual factors, primary stressors, secondary stress, and psychosocial resources associated with each PICS-F impairment at three and six months after hospital discharge?
3) If family satisfaction or caregiver burden is associated with PICS-F impairments, to what extent do they contribute to these outcomes at three and six months after hospital discharge?

Objective: The aim of this study is two-fold, one cross-sectional and one longitudinal aim.
(1) To determine the incidence of each PICS-F impairment (psychological, physical, and cognitive) among family members of ICU survivors of a public hospital in Chile.

(2) To identify factors, during ICU stay and after hospital discharge, associated with the PICS-F impairments among family members of ICU survivors of a public hospital in Chile.

Specific Aims
1. To describe the sociodemographic and clinical features of both family members and ICU patients.

2. To determine the level of anxiety symptoms, depressive symptoms, PTSD symptoms, cognitive impairment, fatigue, perceived social support, resilience, family satisfaction, and caregiver burden among family members of ICU survivors of a public hospital in Chile.

3. To explore the association between contextual factors, primary stressors, secondary stressors, psychosocial resources, and PICS-F impairments among family members of ICU survivors of a public hospital in Chile.

4. To evaluate the extent to which secondary stressors (family satisfaction or caregiver burden) significantly contribute to PICS-F impairments among family members of ICU survivors of a public hospital in Santiago, Chile.

Hypotheses: The proposed hypotheses for the study are:

1. Family members of ICU survivors will experience at least one PICS–F impairment at three and/or six months after hospital discharge.

2. Primary stressors (anxiety, depressive, PTSD symptoms, fatigue, or cognitive impairment) and/or psychosocial resources (social support or resilience) will be associated with at least one PICS – F impairment at three and/or six months after hospital discharge.

3. After controlling for contextual factors, primary stressors, and psychosocial resources, at least one secondary stressor (family satisfaction or caregiver burden) will significantly contribute to the development of at least one PICS – F impairment at three and/or six months after hospital discharge.

SCIENTIFIC NOVELTY

Reduction in mortality rates in the last decades has shifted the attention to PICS, with researchers identifying several challenges related to survivorship. Nonetheless, long-term issues faced by ICU family members remain poorly understood.[70] ICU clinicians and researchers have started to understand that critical illness is not limited to the ICU or hospital setting but is part of the trajectory of a disease that has not been fully explored yet. This study will be one of the first to comprehensively explore all PICS-F impairments simultaneously utilizing the Caregiver Stress Model (CSM).[69] Although Cameron et al.[29] employed the CSM, their study did not include family satisfaction as a secondary stressor, resilience as a psychological resource, and they did not measure PICS-F cognitive impairment. Despite PICS-F awareness, physical and cognitive impairments have been disproportionally less studied. This study will advance knowledge regarding the incidence and factors related to PICS-F physical impairment and will provide initial data on the cognitive impairment that family members of ICU survivors may experience.

Besides, this study will be one of the first to explore how each of the PICS-F impairments is affected by variables and constructs measured during the ICU stay and nearly after ICU discharge. As stated by

Johnson et al.[17] "*… an unknown premorbid functioning could create the inability to distinguish between incident and preexisting symptoms*" observed in PICS-F impairments. The scientific novelty of this study is also grounded in the inclusion of caregiver burden as a secondary stressor that may impact PICS-F impairments. Although evidence on caregiver burden in family members of ICU survivors is emerging, the development of PICS-F impairments may not be solely explained by the residual effect of a traumatic event such as an ICU admission but also, by the burden of providing care to an ICU survivor. Although common issues and mechanisms may be found between caregivers of ICU survivors and caregivers of patients with different diseases (e.g., Alzheimer´s disease, dementia), the acute onset of critical illness and the experience of an ICU stay may significantly impair caregiver's psychological, physical, and mental future resources required to assume the caregiver role after hospital discharge. In other words, the chronic nature and slower progression of patient´s impairments in other caregiver populations may have less impact on caregiver burden by allowing a progressive adaptation of the caregiver to new tasks and strains.

ICU survivorship has imposed a significant burden on the healthcare system, society, and particularly caregivers of those who endure a critical illness. In Chile, around 66% of hospital beds attend poor and middle-class citizens.[71] Therefore, findings from this study conducted in a public hospital will generate data for local healthcare providers such as similar ICUs, hospitals, and the primary care network providing care to family members of ICU survivors. Results from a study also will inform tailored interventions that account for the particular features of the caregiving process of an ICU survivor, particularly the influence of the stage (e.g., ICU hospitalization) that precedes the caregiving role. Finally, this study will provide valuable data on incidence and related factors of PICS-F that are currently unknown, particularly, among Chilean family members of ICU survivors, and will provide data and research experience to conduct larger multicentric studies to validate the findings of this study.

METHODS

Design: This is a quantitative, analytical, and prospective cohort study that considers four times of measurement:

- T_1 → Between the 3rd – 7th day of ICU admission (enrollment)
- T_2 → Nearly after ICU discharge
- T_3 → 3 months after hospital discharge
- T_4 → 6 months after hospital discharge

Setting: The study will be conducted in an adult ICU of a high complexity hospital (> 400 beds) belonging to the Southeast Metropolitan Health Service of Santiago, Chile. This hospital provides care, along with other centers, to a population of around 1.700.000 subjects.[72] This hospital ICU comprises 24 beds with an average of 550 patients admitted during 2019-2020.[73] Visiting policy includes usually 2 hours from Monday to Thursday with flexibility in special cases such as dying patients, collaboration in recovery, or with added caregiving requirements during ICU stay.

Subjects

Inclusion criteria: All adult family members (≥ 18 years old) identified as the patient´s representative, Spanish speakers, and likely to become responsible for providing or coordinating care after hospital discharge will be eligible. Besides, the family member´s ICU patient must have at least 48 hours in the ICU, be > 18 years old, and receive respiratory support (noninvasive ventilation, high-flow nasal cannula, or invasive mechanical ventilation).

Exclusion criteria: Family members of ICU patients with a high impending death risk or likely to be discharged from the ICU in the following 24 hours after screening will be excluded. Family members will be withdrawn at any point of the stud if the patient dies.

Variables: To explore PICS-F impairments incidence and the potential associations with other variables, the following independent variables measured through questionnaires will be used:

- <u>Anxiety and Depressive Symptoms:</u> The Patient Health Questionnaire for Depression and Anxiety (PHQ–4) is an ultra-brief screening tool utilized to explore the occurrence of anxiety and depressive symptoms according to the DSM – IV criteria.[74] PHQ–4 comprises 4 items for cardinal symptoms of both anxiety (2 items) and depressive (2 items) disorders. Answers are scored utilizing a Likert scale ranging from 0 ("Not at all") to 3 ("Nearly every day"). A cut-off value of 6 points for the sum of all items (PHQ-4 ≥ 6) has been utilized to identify subjects with a potential anxiety/depressive disorder.[74] The Chilean-Spanish

version of the PHQ-4 has shown an adequate internal consistency.[26] PHQ-4 will be used in all 4 time points of the study.

- Posttraumatic stress disorder symptoms: The Impact of Event Scale-Revised (IES-R) is a 22-item questionnaire aimed to measure the degree of suffering associated with a traumatic event in the form of subjective stress.[75,76] IES-R items are presented in a Likert scale ranging from 0 ("Not at all") to 4 ("Extremely") and an overall score is calculated by averaging all items. Higher scores reflect a higher presence of PTSD symptoms. The Chilean-Spanish version of the IES-R has shown good reliability.[76] For this study, the 6-item version (IES-6) of the IES-R will be utilized, which comprises items 3, 6, 11, 12, 18, and 21 of the original scale.[77] A cut-off average score of 1.75 (IES-6 ≥ 1.75) has been used to identify family members likely to have a PTSD diagnosis.[25] Since PTSD symptoms are usually measured after the traumatic experience (ICU admission/stay),[7] IES-6 will be applied at T_2, T_3, and T_4.

- Perceived Social Support: The Medical Outcomes Study Social Support Survey (MOS-SSS) was developed to measure the perception of availability of emotional, instrumental, and informational resources by the individual´s social network[78] and includes 18 items with a five-point Likert-type response from 1 ("none of the time") to 5 ("all of the time"). MOS–SSS has shown an adequate internal consistency for its Chilean-Spanish version.[79] For this study, the modified 8-item version (mMOS-SSS) of the MOS-SSS will be utilized, which comprises items 2, 5, 7, 12, 15, 17, 19, and 20 of the original scale.[80] An overall score for the mMOS-SSS is calculated by converting Likert-type responses to a zero to 100 scale and then averaging them. mMOS-SSS will be utilized in all 4 time points of the study.

- Resilience: The Brief Resilient Coping Scale (BRCS) was created to evaluate a subject's propensity to cope with stress in an adaptative way.[81] BRCS is composed of 4 items with Likert responses ranging from 1 ("does not describe me at all") to 5 ("describes me very well"). An overall score can be calculated by summing all items with higher scores reflecting high resilience (range: 4-20). A cut-off value of ≤ 13 points has been established to categorize subjects with low resilience.[81] The Chilean-Spanish version of the BRCS has shown good reliability.[82] Resilience will be measured in all 4 time points of the study.

- Cognitive Impairment (CI): The memory, fluency, and orientation (MEFO) test is a brief screening tool that aims to measure the extent of cognitive deterioration through the assessment of memory, phonemic fluency, and space-time orientation.[83] Compared to the Mini-Mental State Examination, the MEFO test reported having higher discriminatory validity to differentiate between mild CI versus no CI. Overall MEFO test score is 13 points with higher scores reflecting a lower degree of cognitive impairment and subjects with a MEFO < 9 points are classified as with mild cognitive impairment.[83]

- Fatigue: The Short Form Health Survey (SF–36) is a widely used questionnaire to measure health-related quality of life.[84] SF–36 has 8 subscales being the "energy/fatigue" subscale (SF-36 VT) designed to evaluate the subject´s energy, tiredness, and enthusiasm utilizing 4 items on a Likert scale from 1 ("never") to 5 ("always") which are transformed to a 0 – 100 scale and then averaged.[85] Higher scores reflect higher levels of fatigue (or lack of vitality). For this study, a cut-off value < 45 points will be utilized to classify subjects with clinically significant fatigue.[28] The Chilean Spanish version of the SF–36 reported a good internal consistency for both, the global scale and the VT subscale.[86] Fatigue will be measured in all 4 time points of the study.

- Patient´s Dependency: The Barthel Index (BI) is an extensively utilized ordinal scale that aims to evaluate a subject´s functional independence regarding activities of daily living such as dressing, transfer, bathing, feeding, toilet use, and use of stairs.[87] BI includes 10 items, each with 2 to 4 alternatives depending on the case which are scored on a 5 points intervals scale (e.g., 0, 5; 0, 5, 10; 0, 5, 10, 15). An overall score can be obtained by summing all items, with higher scores reflecting higher functional independence. The BI has reported an adequate internal consistency.[88] BI will be completed by the patient´s responsible family member at T_1 regarding the patient's functional independence status before ICU admission. Then, BI will be applied at T_2, T_3, and T_4.

- Family Satisfaction: The Family Satisfaction with Care in the Intensive Care Unit – 24 (FS ICU–24) questionnaire is a widely used tool to explore the family member's evaluation of their ICU experience in terms of communication with ICU health care providers, emotional support, closeness to the patient, ICU environment, decision-making involvement, and quality of nursing care of the patient.[57,89] FS ICU–24 has

24 items with a Likert-type response scale from 1 ("Excellent") to 5 ("Poor"). In this study, the three open-ended questions of FS ICU-24 will not be applied to decrease participant burden. To obtain scores for overall satisfaction, Liker-type responses are transformed to a numeric scale (range: 0–100) and then averaged, with higher scores indicative of higher levels of satisfaction.[89] The Chilean-Spanish version of the FS ICU-24 has shown a high internal consistency.[90] FS ICU-24 will be only applied at T_1 and T_2.

- Caregiver Burden: The Zarit Burden Interview (ZBI) was developed to subjectively measure the burden of caring for an adult with a disabling condition.[91] ZBI is composed of 22-items with a Likert scale extending from 1 ("never") to 5 ("nearly always") with higher scores reflecting a higher level of burden. The Chilean Spanish version of the ZBI has reported an adequate internal consistency.[92] In this study, the 7-item version of the ZBI will be employed which includes items 2, 3, 6, 9, 10, 17, and 22 of the original questionnaire. A cut-off value of ≥ 17 points for the sum of all 7 items has been established to classify subjects with intense caregiver burden.[92] Caregiver burden will be measured only in T_3 and T_4.

All questionnaires in this study are self-reported and the average time for completing them, including the MEFO test, is estimated in 50-55 minutes. Besides, relevant family members' sociodemographic variables plus demographic and clinical data from the patient will be collected at each time point. Detailed information on these variables is presented in the statistical analyses section (Table 1).

Outcome: To determine the presence of each PICS-F impairment, four dichotomous dependent variables will be defined: psychological (PHQ-4 ≥ 6 or IES-6 ≥ 1.75), physical (SF-36 VT < 45), and cognitive (MEFO < 9) impairments at T_3 and T_4. Subjects scoring above or below the respective cut-off value will be categorized as having PICS-F in the respective impairment at T_3 and/or T_4.

Statistical analyses: To explore and characterize data, descriptive statistics will include the analysis of summary statistics and plots (e.g., histograms, q-q plots, and boxplots). Continuous normally distributed variables will be reported using means and standard deviations (SD) and non-normally distributed variables with medians and interquartile range (IQR). Categorical variables will be reported using frequency and percentages. For each questionnaire, internal consistency will be explored calculating Cronbach's alpha.

The incidence of each PICS-F impairments (psychological, physical, and cognitive) at T_3 and T_4, will be determined by classifying subjects according to the proposed cut-off scores. The number of subjects showing a given PICS-F impairment will be divided by the total number of subjects at the specific point of the study. Then, to analyze factors associated with PICS-F impairments, bivariate and multivariate analyses will be conducted. Cross-sectionally, Chi-square or Fisher's exact test will be applied in case of analyzing two or more categorical variables. In the case of two continuous numerical variables, Pearson or Spearman correlation test will be used. If one dependent variable is continuous normally distributed and the independent variable is categorical, then a t-test (two groups) or ANOVA test (three or more groups) will be applied; if the dependent variable is non-normally distributed, Mann-Whitney U test (two groups) or Kruskal-Wallis test (three or more groups) will be applied. Bivariate analyses, along with factors identified in the literature, will help to identify potential independent variables for multivariate models.

Given that this study considers more than one measurement (repeated measures) over time, this information should be considered as correlated within-subjects. Therefore, to analyze variables associated with the PICS-f impairments over time, panel or longitudinal analysis will be used. First, graphical methods, such as panel line plots, will be used to visualize the trend and variation between and within-subjects over time. This analysis will be useful for determining some important covariates and the regression models to be included and applied afterward. Second, the strength of outcome correlation should be defined to decide the regression methods. In this scenario, and considering the nature of the outcome, generalized linear mixed models (GLMM) will be used.[93,94] Contextual factors (family member and patient features) of each subject will be considered fixed effects, but random effects will be included because it is expected that the outcome in each time and for each subject will vary. Given that the outcome is measured as a dichotomous variable, the family will be binomial, and the link function is expected to be logit. Then, the model will be built starting with a large model and trimming it down according to statistical significance but also based on the proposed theoretical model (CSM). The initial models proposed for this study are presented in Table 1.

If family satisfaction (FS) or caregiver burden (CB) is associated with any of PICS-F impairments at T_3 and/or T_4, GLMM with and without the selected secondary stressor will be fitted to analyze the extent of this association. First, FS measured at T_1 will be added to models 1-4 while the FS measure at T_2 will be included in models 5-8. Similarly, CB measured at T_3 will be included in models 9-20 while the T_4 measure of CB will be added to models 17-20. The original model without a secondary stressor will be contrasted

with the same model plus the secondary stressor (either FS or CB). Scores such as Akaike Criterion and likelihood ratio tests will be performed and residual plots will be obtained. Also, 95% confidence intervals will be reported and a *p*-value < .05 will be considered statistically significant. All statistical analyses will be conducted in R, using the applicable packages.

Table 1. Variables and generalized linear mixed models to explore PICS-F impairments

	Independent Variables								Dependent Variables			
**Model #**	**T_1 - ICU admission**								**T_3 & T_4[a]**			
	Patient	*Family member*	*PHQ-4*	*IES-6*	*mMOS-SSS*	*BRCS*	*SF-36 VT*	*MEFO*	*PHQ-4*	*IES-6*	*SF-36 VT*	*MEFO*
1	Age, SOFA, COVID-19 diagnosis, days since ICU admission, BI	Age, gender, education, kinship, MHA, previous ICU experience, income, employment status	X		X	X	X	X	X			
2			X		X	X	X	X		X		
3			X		X	X	X	X			X	
4			X		X	X	X	X				X
	**T_2 - After ICU discharge**								**T_3 & T_4[a]**			
	Patient	*Family member*	*PHQ-4*	*IES-6*	*mMOS-SSS*	*BRCS*	*SF-36 VT*	*MEFO*	*PHQ-4*	*IES-6*	*SF-36 VT*	*MEFO*
5	Age, COVID-19 diagnosis, ICU LOS, BI	Age, gender, education, kinship, MHA, previous ICU experience, income, employment status	X	X	X	X	X	X	X			
6			X	X	X	X	X	X		X		
7			X	X	X	X	X	X			X	
8			X	X	X	X	X	X				X
	**T_3 - 3 months after hospital discharge**								**T_3**			
	Patient	*Family member*	*PHQ-4*	*IES-6*	*mMOS-SSS*	*BRCS*	*SF-36 VT*	*MEFO*	*PHQ-4*	*IES-6*	*SF-36 VT*	*MEFO*
9	Age, COVID-19 diagnosis, ICU and hospital LOS, hospital readmission, discharge location, BI	Age, gender, education, kinship, MHA, previous ICU experience, income, employment status, caregiving hours		X	X	X	X	X	X			
10			X		X	X	X	X		X		
11			X	X	X	X		X			X	
12			X	X	X	X	X					X
									**T_4**			
13			X	X	X	X	X	X	X			
14			X	X	X	X	X	X		X		
15			X	X	X	X	X	X			X	
16			X	X	X	X	X	X				X
	**T_4 - 6 months after hospital discharge**								**T_4**			
	Patient	*Family member*	*PHQ-4*	*IES-6*	*mMOS-SSS*	*BRCS*	*SF-36 VT*	*MEFO*	*PHQ-4*	*IES-6*	*SF-36 VT*	*MEFO*
17	Age, COVID-19 diagnosis, ICU and hospital LOS, hospital readmission, BI, discharge location	Age, gender, education, kinship, MHA, previous ICU experience, income, employment status, caregiving hours		X	X	X	X	X	X			
18			X		X	X	X	X		X		
19			X	X	X	X		X			X	
20			X	X	X	X	X					X

[a] Models will be tested with T_3 and T_4 dependent variables separately. SOFA: sequential organ failure assessment score; ICU: intensive care unit; BI: Barthel index; MHA: mental health antecedents; LOS = length of stay; PHQ-4: patient health questionnaire; IES-6: impact of event scale; mMOS-SSS: modified medical outcomes study social support scale; BRCS: brief resilience coping scale; SF-36 VT: short-form health survey, vitality subscale; MEFO: memory, fluency, and orientation test.

Sample Size: The study sample size will consider the following values: alpha = 5%, a beta = 80%, and precision of estimate = 5%. Because of the lack of studies utilizing the same questionnaires to establish PICS-F incidence, a 20% incidence of PTSD symptoms reported in a study utilizing an IES-6 ≥ 1.75 will be utilized.[25] Based on these parameters, the calculation of the sample size is obtained by the following formula: $[Z^2 \times P \times (1-P)]/e^2$ where *Z* is the value from standard normal distribution associated with a particular confidence interval (CI), which in this case is set at 95%, *P* is the expected true proportion in the population, and *e* is the desire precision level. Consequently, the initial sample size is 247 RFM.

This sample size will be adjusted for a finite population. In this study, the finite population will be 600 since it represents the average 2019-2020 number of ICU admissions (±550) plus a 10% of projected increase during 2023. Data of 2021 will not be considered since it was heavily influenced by the COVID-19 pandemic waves of ICU admissions and it is not likely to be representative of 2023. Then, the adjusted sample size is obtained using the following formula[95]: *(finite population x initial sample size) / (finite population + initial sample size)*. Thus, the adjusted sample size will be 175 RFM. However, considering the attrition rates reported in the literature,[20,21] 40% of losses are contemplated leaving 292 RFM as the final adjusted sample size.

Data collection & enrollment process: Daily, all ICU patients of the unit will be screened by a research assistant (RA) who will verify inclusion/exclusion criteria. After the identification of the patient's representative family member (RFM) with ICU staff, RFM will be approached by the RA during visiting hours to verify additional eligibility criteria and to provide in-depth information regarding the study. If the RFM is interested, the consent form will be signed, and forms and questionnaires will be applied for T_1. Data related to the patients at T_1 and T_2 will be acquired from their medical charts and anonymized for privacy.

After the patient's discharge from the ICU, RFM will be contacted in person or by phone depending on discharge location (e.g., ward, step-down unit, home, assisted living facility), and T_2 questionnaires will be applied. For those ICU patients discharged from the hospital during their ICU stay (e.g., directly to home, assisted living facility, etc.), ICU discharge date will be used as a reference for follow-up interviews after hospital discharge (T_3 & T_4) that will be conducted by phone call. In case of an RFM refers that he/she has no participation in any patient-related caregiving tasks at T_3 and/or T_4, no caregiving-related measures or data will be collected. RFM will provide patient-related data at T_3 and T_4 will. RFM will be withdrawn at any time of the study if the patient dies and only data collected to that date will be analyzed. RFM will receive 5.000 Chilean pesos (USD 6.3) for completing measures at T_2 plus $10.000 (USD 12.5) at T_4 as compensation for the time spent in filling the questionnaires.

The design of the proposed study will consider an average length of stay in the ICU of 9-10 days based on local ICU data of 2019-2020[73] and an expected total hospital LOS of 1 month for each patient, from ICU admission until hospital discharge. In the proposed 24-month recruitment period, an average of 12 RFM should be enrolled monthly (±3 /weekly) to achieve the planned sample size.

Ethical considerations: This study will follow the principles of the Helsinki[96] and Singapore Declaration[97], as well as the applicable laws for conducting research in Chile. All questionnaires utilized in the study are either free to use or the authors were contacted and provided authorization. All participating RFM will sign the informed consent form before any interview or data collection. A waiver of informed consent for the acquisition of patient-related data from medical charts will be requested to the IRB. Patient and RFM-related data will be anonymized to ensure privacy. The PI and the research team will always keep data confidential. Each RFM will be assigned a unique identifier to prevent personal data identification.

Work plan / Gantt chart

	Year 1												Year 2												Year 3											
	1	2	3	4	5	6	7	8	9	10	11	12	13	14	15	16	17	18	19	20	21	22	23	24	25	26	27	28	29	30	31	32	33	34	35	36
Meeting with ANID executives	X						X					X					X					X					X					X			X	
Literature review update		X											X												X											
Establishment of MAEC	X																																			
MAEC meetings*	X	X			X			X		X		X		X		X		X		X		X		X		X		X		X		X	X	X		
Research team meetings		X	X	X	X	X	X	X	X	X	X	X	X	X	X	X	X	X	X	X	X	X	X	X	X	X	X	X	X	X	X	X	X	X	X	X
IRB submission (sponsoring institution)		X																																		
IRB renewal submission														X												X										
IRB submission (hospital)			X																																	
ICU Head physician and head nurse meetings		X											X											X							X					
Study presentation to ICU staff & updates			X												X													X								
Database development/review		X			X			X			X			X			X			X			X			X			X			X			X	X
Recruitment and data collection (T_1)			X	X	X	X	X	X	X	X	X	X	X	X	X	X	X	X	X	X	X	X	X	X	X	X										
ICU discharge follow-up (T_2)			X	X	X	X	X	X	X	X	X	X	X	X	X	X	X	X	X	X	X	X	X	X	X	X	X									
3-month follow-up (T_3)						X	X	X	X	X	X	X	X	X	X	X	X	X	X	X	X	X	X	X	X	X	X	X	X							
6-month follow-up (T_4)									X	X	X	X	X	X	X	X	X	X	X	X	X	X	X	X	X	X	X	X	X	X	X	X				
ANID annual report drafting												X												X												
Results analysis													X											X								X	X			
Congress abstract drafting																					X									X				X		
Manuscript #1 preparation																																X	X	X		
Manuscript #2 preparation																																	X	X	X	
ANID final report drafting																																		X	X	
Dissemination PICS-F seminar																																				X

Note. *IRB = institutional review board; ANID = National Agency of Innovation and Development; RA: research assistants; PI: principal investigators; SC: study coordinator MAEC: Multidisciplinary academic-executive committee; PICS-F: post-intensive care syndrome.

DISSEMINATION: This proposal considers the dissemination of its findings through at least 2 manuscripts of the *WoS Core Collection* journals. Besides, results will be presented in at least 1 international and 2 national congresses. Locally, the main findings of this study will be presented in a seminar aimed to increase awareness regarding PICS-F's impact at the primary care and hospital level. Also, the seminar considers national and international keynote speakers delivering guest lectures. The target population will be ICU clinical staff, primary care and rehabilitation professionals, scientific societies, and relevant stakeholders at the Southeast Metropolitan Health Service and Ministry of Health.
